# Prenatal Maternal Stress Exacerbates Experimental Colitis of Offspring in Adulthood

**DOI:** 10.3389/fimmu.2021.700995

**Published:** 2021-11-03

**Authors:** Yue Sun, Runxiang Xie, Lu Li, Ge Jin, Bingqian Zhou, Huan Huang, Mengfan Li, Yunwei Yang, Xiang Liu, Xiaocang Cao, Bangmao Wang, Wentian Liu, Kui Jiang, Hailong Cao

**Affiliations:** ^1^Department of Gastroenterology and Hepatology, General Hospital, Tianjin Medical University, Tianjin, China; ^2^Institute of Digestive Diseases, Tianjin Key Laboratory of Digestive Diseases, Tianjin, China

**Keywords:** early life, prenatal maternal stress, microbiota, mucosal barrier, colitis

## Abstract

The prevalence of inflammatory bowel disease (IBD) is increasing worldwide and correlates with dysregulated immune response because of gut microbiota dysbiosis. Some adverse early life events influence the establishment of the gut microbiota and act as risk factors for IBD. Prenatal maternal stress (PNMS) induces gut dysbiosis and perturbs the neuroimmune network of offspring. In this study, we aimed to investigate whether PNMS increases the susceptibility of offspring to colitis in adulthood. The related index was assessed during the weaning period and adulthood. We found that PNMS impaired the intestinal epithelial cell proliferation, goblet cell and Paneth cell differentiation, and mucosal barrier function in 3-week-old offspring. PNMS induced low-grade intestinal inflammation, but no signs of microscopic inflammatory changes were observed. Although there was no pronounced difference between the PNMS and control offspring in terms of their overall measures of alpha diversity for the gut microbiota, distinct microbial community changes characterized by increases in *Desulfovibrio*, *Streptococcus*, and *Enterococcus* and decreases in *Bifidobacterium* and *Blautia* were induced in the 3-week-old PNMS offspring. Notably, the overgrowth of *Desulfovibrio* persisted from the weaning period to adulthood, consistent with the results observed using fluorescence *in situ* hybridization in the colon mucosa. Mechanistically, the fecal microbiota transplantation experiment showed that the gut microbiota from the PNMS group impaired the intestinal barrier function and induced low-grade inflammation. The fecal bacterial solution from the PNMS group was more potent than that from the control group in inducing inflammation and gut barrier disruption in CaCo-2 cells. After treatment with a TNF-α inhibitor (adalimumab), no statistical difference in the indicators of inflammation and intestinal barrier function was observed between the two groups. Finally, exposure to PNMS remarkably increased the values of the histopathological parameters and the inflammatory cytokine production in a mouse model of experimental colitis in adulthood. These findings suggest that PNMS can inhibit intestinal development, impair the barrier function, and cause gut dysbiosis characterized by the persistent overgrowth of *Desulfovibrio* in the offspring, resulting in exacerbated experimental colitis in adulthood.

## Introduction

Inflammatory bowel disease (IBD), comprising ulcerative colitis and Crohn’s disease, involves a group of chronic, recurrent, and destructive intestinal inflammatory conditions (1). The etiology and pathogenesis of IBD have not been entirely expounded yet. IBD is becoming highly prevalent worldwide, showing pronounced growth in newly industrialized countries (2) and posing a challenge to health services. Potential factors that contribute to these transitions of incidence and prevalence include the urbanization, industrialization, and westernization of society (3). Stress has become a sizeable component of modern lifestyle and greatly contributes to the increased prevalence of IBD. The importance of stress on IBD has long been recognized in the literature (4, 5). Stress triggers the flares of patients with IBD (6–11), and the underlying mechanisms include the dysregulation of the gut–brain–microbiome axis and the neuroendocrine system (12, 13).

The early life stage is a pivotal period of developmental plasticity. Recent studies have emphasized maternal influences on fetuses and indicated that early life events (such as the delivery mode, breastfeeding, and antibiotic exposure) can affect the potential risk of IBD (14–17). According to available data, initial intestinal microbial colonization appears immediately after birth (18, 19). Establishment of the intestinal microflora, which occurs in early life, known as the window of opportunity, is of paramount importance to the maturation of the immune system. One hypothesis is that the disruption of the microbiome during the crucial window can aggravate a disease later in life in a manner that influences childhood development and causes long-lasting detrimental consequences of immunopathology (20–22). Neonatal colonizers are mainly derived from maternal microbiota, such as the gut microbiota, vaginal microbiota, and breast milk (23). Perinatal factors, such as nutrition, antibiotic use, and maternal stress, alter the maternal gut microbiota during pregnancy and shape the establishment of the early microbiome in neonates (24, 25).

Growing evidence has suggested that early life adversities, including prenatal and postnatal events, can alter the gut microbiota and make it potentially prone to certain diseases (26–29). Prenatal maternal stress (PNMS) induces gut microbiota dysbiosis, perturbs the neuroimmune network, and upregulates the levels of placental inflammatory factors, and may raise susceptibility to asthma, visceral hypersensitivity, and neurological disorders in offspring through vertical transmission (30–34). In fact, some clinical studies demonstrated that maternal prenatal stress is associated with the composition of infant fecal microbiota (35, 36). Repeated restraint stress from the 14th to the 20th day of embryo development can decrease the density of distal colonic innervation and change the gut microbiota of offspring rats (37). Prenatal stress in early pregnancy leads to changes in the gut niche, transcriptome, and immune cells in the offspring. Furthermore, these changes are linked to alterations in the adult intestinal permeability and microbiota following additional stress in adulthood (38). However, whether or not PNMS increases the susceptibility to IBD in the next generation and what mechanisms are involved are unclear.

Here, we investigated the effects of PNMS on the susceptibility of offspring to colitis in adulthood. We show that PNMS impaired the intestinal development and barrier function in the offspring. We further provide evidence that PNMS can disturb the establishment of the intestinal microflora, induce the persistent overgrowth of *Desulfovibrio*, and engender low-grade colonic inflammation in the offspring. Interestingly, dysbiosis persisted into adulthood, where experimental colitis worsened. Our findings demonstrated that PNMS, one of the common adverse early life events, has negative effects on the intestinal development and microbiota establishment of offspring, thus raising the risk of colitis. The study is of pivotal importance to the study of the pathogenesis of IBD.

## Materials and Methods

### Animals and Prenatal Maternal Stress

Experiments were performed on C57BL/6 mice (provided by Peking Union Medical College). The animals were kept in specific pathogen-free conditions and allowed to acclimatize for 1 week before experimentation. They were fed with an AIN-93M rodent diet and sterile filtered water in a controlled environment under a 12-h light/12-h dark schedule. Mice were mated overnight; a positive vaginal plug was denoted as embryonic day 0 of pregnancy.

**Figure 1:**
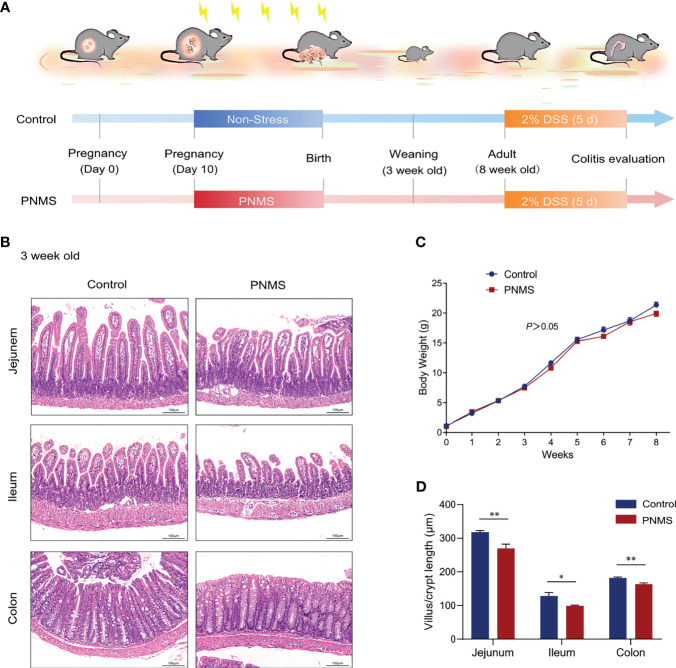
Prenatal maternal stress impaired the intestinal development of 3-week-old offspring. **(A)** Flow diagram of the experimental procedure. **(B)** Microscopic observation of the intestinal and colonic development of 3-week-old pups with hematoxylin and eosin (HE) staining. **(C)** Changes in the body weight of offspring mice from week 0 to week 8. **(D)** Measurement of the villus lengths in the jejunum and ileum and the crypt depths of the colon. *PNMS*, prenatal maternal stress; *DSS*, dextran sulfate sodium. *Scale bar*, 100 μm. Magnification, ×200. *p < 0.05, **p < 0.01. PNMS (*n* = 6) *vs.* control (*n* = 6).

A brief description of the experimental flow is shown in **Figure 1A**. Pregnant dams were randomly assigned to either the control (*n* = 6) or the PNMS (*n* = 6) group and housed individually. Beginning on E10, pregnant dams assigned to the PNMS group were subjected to variable stress, and the paradigm was modified from the previous study (39). Mice randomly experienced two of the following stressors daily to prevent habituation to stress: constrained inside a 50-ml tube for 60 min, wet bedding with saturated water overnight, lights on during the dark phase, 1 h white noise generated by a speaker (2–20 kHz, 90 dB), and 60 min water avoidance stress. Mice were not subjected to the same stressor for two consecutive days. The morning on which the litter was found was considered postnatal day (PND) 0. On PND 1, litter size was standardized to four per litter. In the 21 days between birth and weaning, the mice were housed with their mother and littermates. On PND 22, one mouse from each litter was randomly selected and sacrificed. The remaining pups were weaned in the same-sex housing until the end of the assay (three or four in each cage). Offspring was weighed weekly after birth. The Ethics Committee of Tianjin Medical University (Tianjin, China) approved all the animal procedures.

### Tissue Collection

Offspring mice 3 and 8 weeks old were euthanized. Small intestine and colon tissues were immediately removed and washed with pre-cooled phosphate-buffered saline (PBS). The small bowel was separated into three segments. For the preparation of paraffin slices, distal tissues were Swiss rolled and fixed with 4% paraformaldehyde. The sections were subsequently used in multiple staining experiments. The proximal tissues of four intestinal segments were collected and placed in a −80°C refrigerator after snap freezing in liquid nitrogen.

### Histology and Immunohistochemistry

Paraffin-embedded specimens were sectioned at a thickness of 4 μm and stained with hematoxylin and eosin (HE). For the histological assessment of intestinal development, the villus height and crypt depth of each intestinal section were measured under a light microscope (DM5000 B; Leica, Wetzlar, Germany), and 100 well-oriented, intact villus/crypt of each mouse were randomly selected for evaluation.

For immunohistochemistry, tissue microarray sections were deparaffinized with xylene, rehydrated with gradient concentrations of ethanol, and soaked in 3% hydrogen solution. Then, the sample was exposed to antigen hot retrieval with citrate buffer and further incubated with a blocking serum. After blocking, the tissue slices were incubated overnight with a monoclonal anti-Ki67 (ab16667; Abcam, Cambridge, UK), anti-Muc2 (Santa Cruz Biotechnology, Inc., Santa Cruz, CA, USA), or an anti-lysozyme (ab108508; Abcam) antibody. After incubation, the sections were washed with PBS, incubated with labeled polymer–horseradish peroxidase, and counterstained with 3,3′-diaminobenzidine. The sections were observed under a Leica microscope (DM5000 B; Wetzlar, Germany). Positive cells were counted by observing 100 villi or crypts per mouse from at least five randomly selected fields in a blinded manner. The original magnification of the HE and immunohistochemical staining was ×200.

### Immunofluorescent Staining

Small intestine and colon sections (4 µm thick) were deparaffinized and hydrated and then subjected to heat-induced antigen retrieval. PBS with 5% bovine serum albumin (BSA) (Solarbio, Beijing, China) was used as the blocking agent for nonspecific binding. Thereafter, the slides were incubated with an anti-immunoglobulin A (IgA) antibody (ab223410; Abcam) overnight. Then, secondary antibody conjugates, DyLight 488 anti-goat immunoglobulin G (IgG) secondary antibody (Abbkine, Wuhan, China) or anti-rabbit IgG (H&L) secondary antibody, Alexa Fluor^®^ 488 conjugate (Invitrogen, Carlsbad, CA, USA), were added to the sections for 60 min (avoiding light). Finally, 4,6-diamidino-2-phenylindole (DAPI; Solarbio, Beijing, China) marked nuclei blue. Immunofluorescence pictures were obtained under a Pannoramic MIDI II slide scanner (3DHISTECH, Budapest, Hungary) at ×200 magnification. As described previously, the average quantity of positive cells in 100 villi was recorded per mouse. For zonula occludens-1 (ZO-1) immunofluorescence staining, the cells were fixed with 4% (*w*/*v*) paraformaldehyde solution for 15 min and permeabilized with 0.2% (*w*/*v*) Triton X-100 for 10 min. Then, the cells were blocked for 1 h in 10% donkey serum and incubated with anti-ZO-1 (61-7300; Thermo Fisher Scientific, Waltham, MA, USA) overnight at 4°C. Alexa 488-conjugated donkey anti-rabbit IgG and DAPI were incubated with cells separately. Images were obtained at a magnification of ×400.

### Intestinal Permeability Measurement

To assess intestinal permeability, the absorption of fluorescein isothiocyanate–dextran (FITC-D) (MW: 4,000 Da; Sigma-Aldrich, St. Louis, MO, USA) from the gut lumen into the plasma was monitored. Mice received FITC-D (6 mg/10 g body weight, 50 mg/ml) through oral gavage for 4 h prior to blood collection. The blood was drawn from the ophthalmic vein and collected with heparinized microcentrifuge tubes. Serum samples were separated through centrifugation at 2,000 rpm for 10 min. The fluorescence intensity of each sample was measured (excitation = 485 nm, emission = 530 nm). The FITC-D concentration was derived from an FITC-D standard curve produced through serial dilution in mouse plasma.

### Periodic Acid–Schiff Staining

Colon sections were incubated in 1% periodic acid solution for 10 min, washed, incubated in Schiff’s reagent for 40 min, washed again, and counterstained with hematoxylin solution for 5 min. Between each step, the samples were rigorously washed with 1× PBS buffer. Positive cells were calculated by observing 100 villi or crypts per mouse from at least five randomly selected fields in a blinded manner. Images were taken with a Leica microscope (DM5000 B; Wetzlar, Germany) at ×200 magnification.

### Real-Time PCR Analysis

The extraction of total RNA from the tissues was performed using an RNeasy Mini Kit (Qiagen, Carlsbad, CA, USA), and then reverse transcription was conducted with a TIANScript Reverse Transcription Kit (TIANGEN Inc. Beijing, China). Quantitative PCR analyses in real time were carried out using a StepOnePlus Real-Time PCR apparatus (Applied Biosystems, Carlsbad, CA, USA) according to the manufacturer’s directions. The PCR procedure was performed as follows: incubation (95°C, 10 min), 40 cycles of denaturation (95°C, 15 s), and annealing and extension (60°C, 1 min). Quantification of amplicons was accomplished with SYBR Green fluorescence. The oligonucleotide primer pairs included ZO-1, claudin 3, occludin, Muc2, cryptdin, regenerating islet-derived protein (Reg3γ), interleukin 1 beta (IL-1β), tumor necrosis factor alpha (TNF-α), interferon gamma (IFN-γ), transforming growth factor beta (TGF-β), IL-6, and IL-10, which were synthesized by GENEWIZ, Inc. (Beijing, China). The sequences are summarized in **Supplementary Table S1**. Relative messenger RNA (mRNA) quantification was calculated using the 2^−ΔΔCT^ method.

### Western Blotting

Total protein extracts of the colon tissues were harvested with RIPA buffer (Solarbio, Beijing, China). A bicinchoninic acid protein assay kit (Thermo Fisher Scientific) was used in evaluating the protein concentration and homogenizing proteins. Protein samples were loaded onto an SDS-PAGE system for separation and subsequently electrotransferred onto PVDF membranes (Invitrogen). After blocking with BSA, the membranes were incubated with appropriate primary antibodies against β-actin (anti-mouse) and anti-CLDN3 (anti-mouse) and then with horseradish peroxidase (HRP)-conjugated secondary antibodies (all from Cell Signaling Technology, Danvers, MA, USA). An enhanced chemiluminescence (ECL) chromogenic substrate was used in visualizing bands. Proteins were quantified using the ImageJ plot profile tool.

### 16S rDNA Amplicon Sequencing

Offspring stool specimens were detected at 3 and 8 weeks after birth. Bacterial DNA extraction, 16S ribosomal DNA (rDNA) amplicon sequencing, and 16S rDNA gene analysis were conducted by the Realbio Genomics Institute (Shanghai, China). The QIAamp^®^ Fast DNA Stool Mini Kit (QIAGEN, Hilden, Germany) was used in isolating total genomic bacterial DNA. Bacterial 16S ribosomal RNA (rRNA) genes (V3–V4 regions) were amplified using genomic DNA as the template on a thermal cycler, and specific primers were used (forward: 5′-TCGTCGGCAGCGTCAGA TGTGTATAAGAGACAGCCTACGGGNGGCWGCAG-3′; reverse: 5′-GTCTCGTGGGCTCGGAG ATGTGTATAAGAGACAGGACTACHVGGGTATCTAATCC-3′). PCR was conducted using the high-fidelity enzyme of a KAPA HiFi Hotstart PCR kit to maintain the accuracy and efficiency of amplification. After amplification, purification, and quantification of the fragments, the DNA sample was produced using the Illumina HiSeq™ 2000 platform. The chimeras and singletons were removed, and the operational taxonomic units (OTUs) were obtained at 97% sequence similarity by clustering with USEARCH (v7.0.1090) in the QIIME pipeline. A comparison of the RDP database with the Basic Local Alignment Search Tool (BLAST) program was performed for the analysis of classification. Diversity measure and rank abundance curve analyses were conducted using the abundance data in QIIME. The resulting matrix of distances was obtained through principal coordinate analysis (PCoA) between groups. Differences between and within groups were compared through analysis of similarities (ANOSIM). Abundance and diversity were estimated using the Shannon and Simpson indexes. Linear discriminant analysis (LDA) score histogram and cladogram were created through linear discriminant analysis effect size (LEfSe) analysis. Spearman’s correlations were then calculated for the differential bacterial and environmental predictors. The raw sequencing data were uploaded to NCBI (SRA accession: NCBIPRJNA728691).

### Fluorescence *In Situ* Hybridization

Fluorescence *in situ* hybridization (FISH) was performed as described previously (40). 16S rRNA-targeted oligonucleotide probes were obtained from probeBase (http://www.microbial-ecology.net/probebase/). *Desulfovibrio* Cy5-conjugated specific probe (ATGTTATCCATGTGTATAGGGC) was labeled with Spectrum-Red (Thermo Fisher Scientific). An FITC-conjugated EUB338 universal bacterial probe (GCTGCCTCCCGTAGGAGT), as the positive control, was labeled with Spectrum-Green (Thermo Fisher Scientific). In brief, 5-µm-thick colon paraffin sections were pretreated and hybridized with the *Desulfovibrio* and EUB338 probes for 5 h. Then, DAPI (Thermo Fisher Scientific) was added after incubation, and the nucleus was stained and observed under a fluorescence microscope (magnification, ×400).

### Fecal Microbiota Transplantation

Fresh stool samples (600 mg) were collected from the control and 3-week-old PNMS offspring. In an anaerobic environment, fecal pellets from each group were pooled, homogenized, and suspended in 15 ml of old sterile PBS solution with 0.05% cysteine hydrochloride. After standing for 2 min, the supernatant was collected, transferred to sterile Hungate anaerobic tubes, and stored at −80°C. Eight-week-old mice were divided into two groups [fecal microbiota transplantation (FMT)-Control and FMT-PNMS groups] containing five mice each. The FMT-Control group received fecal transplantation from the control offspring, and the FMT-PNMS group received fecal transplantation from the PNMS offspring. For the ablation of commensal microbiota, the mice received an antibiotic cocktail of ampicillin (200 mg/L), metronidazole (200 mg/L), neomycin (200 mg/L), and vancomycin (200 mg/L) *via* drinking water as described in the literature (41). After 5 days of the mice receiving the antibiotic cocktails, a 200-µl bacterial solution was gavaged to each mouse three times a week for 4 weeks (**Figure 5A**).

### Cell Culture and Treatment

Human colonic epithelial Caco-2 cells were cultured in Minimum Essential Medium (MEM; Gibco, Carlsbad, CA, USA) supplemented with 20% FBS (Gibco) and 1.0% non-essential amino acid. This test starved the Caco-2 cells with 0.5% serum medium for 12 h when they had grown to 70%–80% confluence. After starvation, the Caco-2 cells were incubated with fecal bacterial solution (1:200) from the control and 3-week-old PNMS offspring separately for 3 h. Then, the medium was changed to a medium with 0.5% serum and 1% penicillin–streptomycin, and the cells were harvested for experiments 12 h after co-culture with bacterial solution. For the antagonist studies, the cells were incubated with adalimumab (10 μg/ml; Abbott, Chicago, IL, USA) after the addition of fecal bacterial fluid at the end of the experiment. All the cells in each group were collected for the extraction of total protein and RNA.

### Experimental Colitis Model

Eight-week-old adult mice were stochastically assigned through feeding with 2% (*w*/*v*) dextran sulfate sodium (DSS, 36–50 kDa; MP California, USA) solution or water for 5 days to build a colitis model. During the modeling process, the DSS solutions were refreshed daily. We used the disease activity index (DAI), colonic length, spleen weight, and histological change in assessing the severity of colitis. The DAI scoring system was based on weight loss, rectal bleeding, and stool consistency, as described previously (42). The entire colon (from the cecum to the anus) was removed, and the length was measured and recorded. The histological score of the degree of colitis injury was measured with an orderly categorical measurement ranging from 0 to 40 (43). Scoring was performed by multiplying three histological features, namely, extent of inflammatory cell infiltration (0–3), depth of lesions (0–3), and damage of the crypt (0–4), by the corresponding percentage of the affected region (1–4). Histopathological assessment of the colon samples was performed in a blinded manner by a pathologist.

### Statistical Analysis

Statistical calculations were conducted with GraphPad Prism (version 8.0). The measurement data were described as the mean ± standard error of the mean (SEM). A *t*-test or the Mann–Whitney *U* test was used between two groups, whereas ANOVA was used among four groups. Statistical significance was set at *p* < 0.05.

## Results

### PNMS Inhibited Intestinal Development of the 3-Week-Old Offspring

The offspring from the PNMS and control groups followed a similar weight curve during the first 8 weeks of life (**Figure 1C**). Before weaning, the growth of the offspring is mainly supported by breastfeeding and parental care provided by mammalian mothers. Moreover, the gastrointestinal tracts of rodents mature in the third postnatal week. Therefore, the weaning period was used as a key node for the evaluation of maternal influence on the development of offspring in this study. To test whether or not PNMS affects intestinal growth, the villus length in the jejunum and ileum and the crypt depth in the colon were observed through HE staining. Morphometric quantification showed a significant decrease in the average villus length/crypt depth in PNMS mice relative to the control group (**Figures 1B, D**). Similar differences in the intestinal villus structure were not observed in adulthood (data not shown).

**Figure 2:**
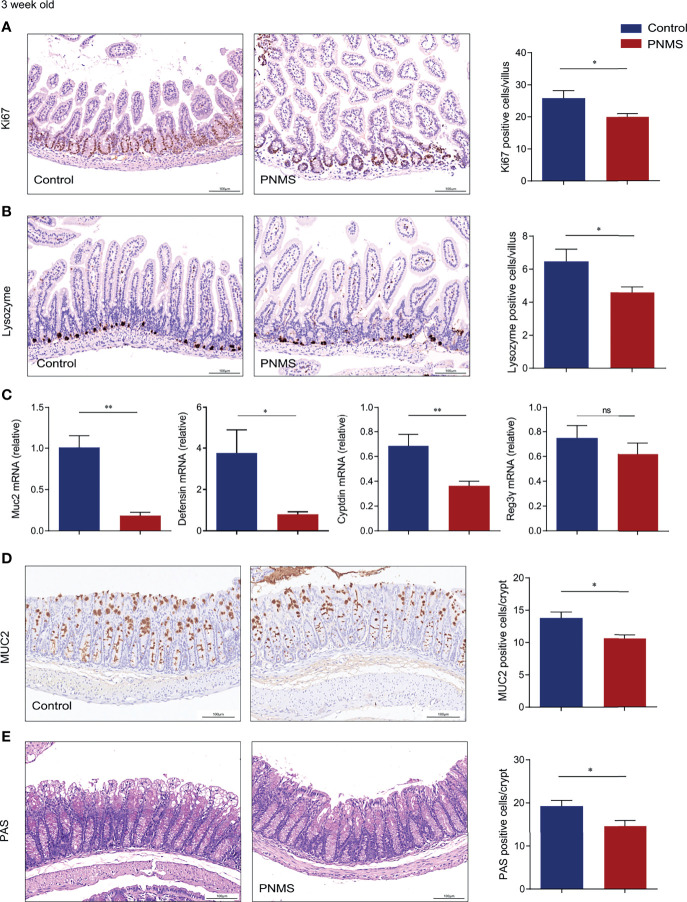
Prenatal maternal stress inhibited the proliferation and differentiation of the intestinal tract in 3-week-old offspring mice. **(A)** Cell proliferation in the small intestine was assessed through Ki-67 immunohistochemical staining. **(B)** Paneth cells in the small intestine were evaluated by lysozyme immunohistochemical staining and the quantification of positively stained cells. **(C)** Relative mRNA expression levels of Muc2 in the colon and defensin, cryptdin, and Reg3γ in the small intestine. **(D)** Immunohistochemical staining for MUC2 mucin (*brown*) in colon tissue section. **(E)** Periodic acid–Schiff (PAS) staining for goblet cells in colon tissue. Quantification of positive cells per villus as shown by the histogram. At least 100 villi were randomly selected for each sample for calculation. *PNMS*, prenatal maternal stress. *Scale bar*, 100 μm. Magnification, ×200. *p < 0.05, **p < 0.01, ^ns^*p* > 0.05. PNMS (*n* = 6) *vs.* control (*n* = 6).

The intestinal epithelium continuously self-renews throughout the cell proliferation and differentiation cycles. Differentiated enterocytes arise from intestinal stem cells residing in the crypt region. Here, we evaluated the impact of PNMS on intestinal epithelial cell proliferation and differentiation in 3-week-old mice. The average count of Ki67-positive cells decreased in PNMS pups (**Figure 2A**) compared with that in the control group. Lysozyme immunohistochemical staining suggested a lower count of Paneth cells in PNMS mice (**Figure 2B**). As a critical component of intestinal resistance to infection by enteropathogenic bacteria, Paneth cells secrete antimicrobial peptides, such as cryptdin, defensin, and Reg3γ. Real-time PCR data showed that the mRNA expressions of cryptdin and defensin decreased in PNMS pups (**Figure 2C**). In addition, in comparison with the control pups, PNMS pups showed an obvious decreasing trend with regard to the number of MUC2-positive cells and periodic acid–Schiff (PAS)-positive cells, indicating cell differentiation (**Figures 2D, E**). Meanwhile, Muc2 gene expression in PNMS pups was lower than that in the control pups (**Figure 2C**). These results showed that PNMS has an adverse effect on the intestinal development of offspring.

### PNMS Disrupted the Gut Barrier Function of Offspring

**Figure 3:**
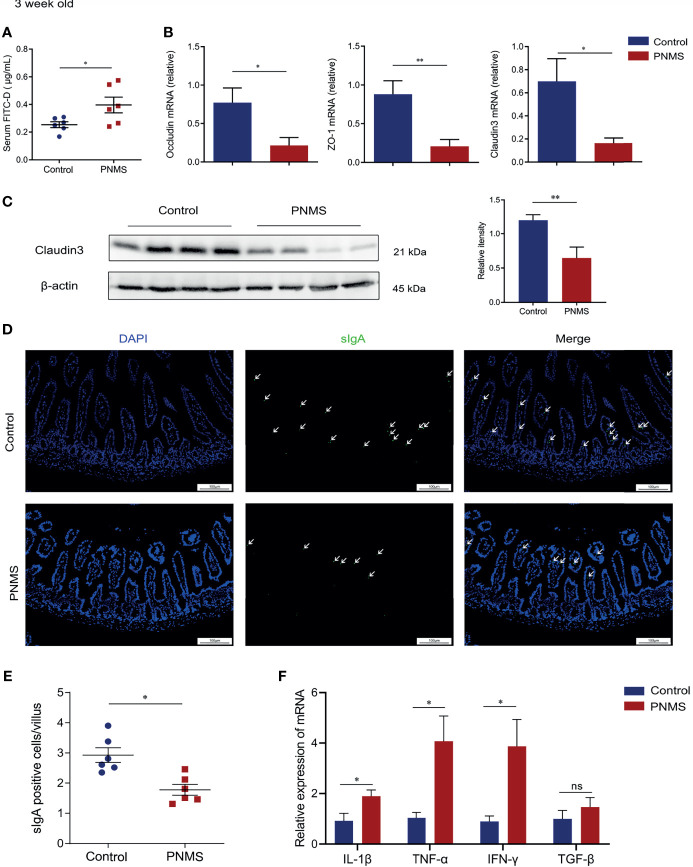
Prenatal maternal stress impaired the intestinal barrier and induced colonic mucosal low-grade inflammation in 3-week-old offspring. **(A)** Intestinal permeability was measured through fluorescein isothiocyanate–dextran (FITC-D) assay. **(B)** Quantification of the relative mRNA expression levels of ZO-1, occludin, and claudin 3. **(C)** Western blot bands and relative quantification of claudin 3 in the colon. **(D)** Immunofluorescence staining of sIgA (*green*) and nuclear staining with DAPI (*blue*) were visualized *via* a fluorescent microscope. **(E)** Number of sIgA-positive cells per intestinal villus. **(F)** mRNA levels of the inflammatory cytokines in the colon. *PNMS*, prenatal maternal stress; *sIgA*, secretory immunoglobulin **(A)** *Scale bar*, 100 μm. Magnification, ×200. *p < 0.05, **p < 0.01, ^ns^*p* > 0.05. PNMS (*n* = 6) *vs.* control (*n* = 6).

The gut barrier is crucial to the maintenance of homeostasis in the gut mucosa. Barrier destruction underlies mucosal inflammation in IBD. Here, the intestinal barrier was evaluated at 3 weeks of age. The concentration of FITC-D in the serum was significantly attenuated in PNMS mice, indicating elevated intestinal epithelial permeability and disrupted intestinal integrity (**Figure 3A**). Tight junctions represent an essential feature of the first defense barrier of the intestine. To further investigate changes in the gut barrier, we evaluated the expressions of the tight junction proteins. The expression levels of ZO-1, claudin 3, occludin, and ZO-1 mRNA were reduced in PNMS mice (**Figure 3B**) compared with those in the control mice. Western blot results showed lower claudin 3 protein levels detected in PNMS mice (**Figure 3C**).

Intestinal secretory immunoglobulin A (sIgA) is a main component of the defense line, preventing pathogenic mucosal adhesion and colonization. We measured the expression of intestinal IgA in the small intestine at the IgA enrichment intestinal tract in 3-week-old pups. Immunofluorescence staining demonstrated that the number of sIgA-positive cells (emphasized by a white arrow in the figure) decreased in the lamina propria of the offspring exposed to PNMS (**Figures 3D, E**). Collectively, PNMS can impair not only the mechanical barrier but also the chemical and immune barriers.

### PNMS Induced Low-Grade Intestinal Inflammation in 3-Week-Old Offspring

No microscopic inflammation was observed in the HE-stained sections of the intestinal tissues of 3-week-old pups. Then, we examined the relative expressions of colonic pro-inflammatory cytokines through real-time PCR. The data showed that the mRNA expression levels of IL-1β, IFN-γ, and TNF-α were upregulated in PNMS mice, whereas that of TGF-β did not reach statistical difference (**Figure 3F**). These observations indicated that PNMS in early life induces low-grade inflammation in offspring.

### PNMS Induced Overgrowth of *Desulfovibrio* in 3-Week-Old Offspring

**Figure 4:**
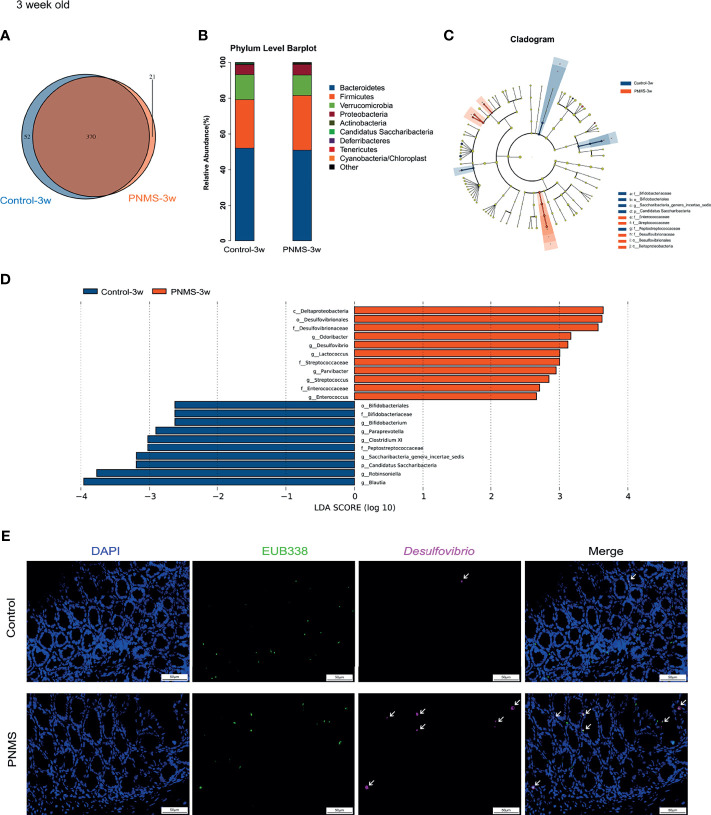
Prenatal maternal stress induced dysbiosis of the gut microbiota in 3-week-old offspring. **(A)** Venn diagram of the operational taxonomic units (OTUs). **(B)** Microbial composition at the level of bacterial phylum. **(C, D)** Cladogram and the linear discriminant analysis (LDA) scores showed significant bacterial differences between the two groups by linear discriminant analysis effect size (LEfSe). **(E)** Fluorescence *in situ* hybridization (FISH) detection of *Desulfovibrio* in colon tissues of the control and PNMS offspring using a fluorescein isothiocyanate (FITC)-conjugated universal bacterial 16S rDNA-directed oligonucleotide probe (EUB338, *green*) and Cy5-conjugated *Desulfovibrio* specific probe (*red*). *PNMS*, prenatal maternal stress. *Scale bar*, 50 μm. Magnification, ×400. Control-3w (*n* = 15) *vs.* PNMS-3w (*n* = 15).

To determine whether or not exposure to PNMS results in alterations of the composition of the microbiota, we collected the feces of 3-week-old pups for 16S rDNA sequencing. The overlap and difference of OTUs in the fecal microbiota between the two groups were displayed in a Venn diagram (**Figure 4A**). A total of 391 OTUs were obtained from PNMS mice, and 422 OTUs were obtained from control mice, sharing 370 OTUs.

Next, we characterized the bacterial communities to compare the community structure and diversity at different levels. Bacterial composition was assessed at different taxonomic levels (**Figure 4B** and **Supplementary Figures S1A, B**). The relative abundance of the bacterial phyla between the two groups was similar, and no statistical difference was found (**Figure 4B**). The composition of the gut microbiota at the level of bacterial phylum in each specimen is shown in **Supplementary Figure S1C**. No significant difference in the alpha diversity was observed (**Supplementary Figure S1D**). Bacteria with significant differential abundance between the control and PNMS groups were detected (**Figures 4C, D** and **Supplementary Figures S1E, F**). Notably, Desulfovibrionales, Desulfovibrionaceae, and *Desulfovibrio*, which are pro-inflammatory bacteria, increased in the PNMS group. In addition, other potential Gram-positive coccus pathogens, such as *Streptococcus* and *Enterococcus*, increased in the PNMS group. In contrast, Bifidobacteriales, Bifidobacteriaceae, and *Bifidobacterium*, which are growth-promoting and anti-inflammatory probiotics, dramatically decreased in the PNMS group. In parallel, short-chain fatty acid (SCFA)-producing bacteria, such as *Blautia* and *Robinsoniella*, showed significantly reduced abundance in mice exposed to PNMS. Two-color FISH further showed that *Desulfovibrio* was enriched in the colon of PNMS (**Figure 4E**). In-depth analysis revealed a correlation between intestinal microbiota and inflammation. Some specific genera of bacteria (especially *Desulfovibrio*) were positively correlated with intestinal inflammatory factors (**Figure 8B**).

In summary, these findings indicated that exposure to PNMS alters the composition of the intestinal microbiota and, thus, influences the establishment and development of commensal microflora in early life.

### Gut Microbiota From the PNMS Group Impaired Intestinal Barrier Function and Induced Low-Grade Inflammation

**Figure 5:**
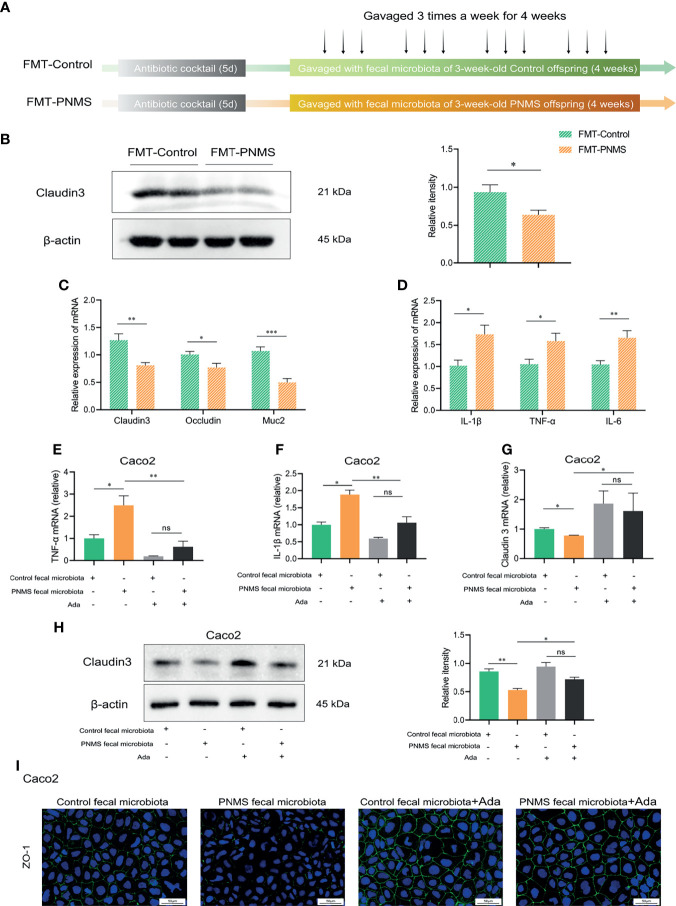
Fecal microbiota from PNMS offspring impaired the gut barrier function and induced low-grade inflammation. **(A)** Experimental workflow. **(B)** Detection of claudin 3 protein by Western blotting. **(C)** Quantification of the relative mRNA expression levels of claudin 3, occludin, and Muc2. **(D)** mRNA levels of the inflammatory cytokines in the colon. **(E–I)** Treatment of Caco-2 cells with the fecal bacterial solution from the control and 3-week-old PNMS offspring mice alone or in combination with adalimumab. **(E, F)** Expressions of TNF-α and IL-1β assessed by quantitative PCR (qPCR). **(G, H)** Expression of claudin 3 assessed by Western blot and qPCR. **(I)** Fluorescence images for ZO-1. *FMT*, fecal microbiota transplantation; *PNMS*, prenatal maternal stress; *Ada*, adalimumab. *Scale bar*, 50 μm. Magnification, ×400. *p < 0.05, **p < 0.01, ***p < 0.001. FMT-control (*n* = 5) *vs.* FMT-PNMS (*n* = 5). ns, P < 0.05.

Through the FMT experiment, we found that the gut microbiota from the PNMS group impaired the intestinal barrier function and induced low-grade inflammation. The fecal bacterial solution from the PNMS offspring significantly reduced the protein expression of claudin 3 and the relative mRNA levels of claudin 3, occludin, and Muc2 in the colon tissues more potently than did the fecal bacterial solution from the control offspring (**Figures 5B, C**). Furthermore, a subsequent increase in the expressions of the inflammatory factors IL-1β, TNF-α, and IL-6 was observed in the colon of FMT-PNMS mice (**Figure 5D**). These results suggested that the gut microbiota plays important roles in PNMS activities that affect the intestinal health of offspring. In this *in vitro* assay, the fecal bacterial solution was added to the Caco-2 culture. The results showed that the fecal bacterial solution from the PNMS group was more potent than that from the control group in inducing inflammatory cytokine expression and gut barrier disruption in Caco-2 cells (**Figures 5E–I**). The relative mRNA levels of TNF-α and IL-1β were upregulated, and the relative mRNA and protein levels of claudin 3 were downregulated (**Figures 5E–H**). The fluorescence intensity of ZO-1 in the PNMS group was lower than that in the control group (**Figure 5I**). We then determined whether or not adalimumab can reverse this interaction. The results showed that adalimumab can partially rescue the inflammatory and barrier disruption phenotype (**Figures 5E–I**). No statistical difference in the relative mRNA levels of TNF-α and IL-1β and the relative mRNA and protein levels of claudin 3 was found between the two groups of adalimumab.

### Overgrowth of *Desulfovibrio* Induced by PNMS Continued in 8-Week-Old Offspring

**Figure 6:**
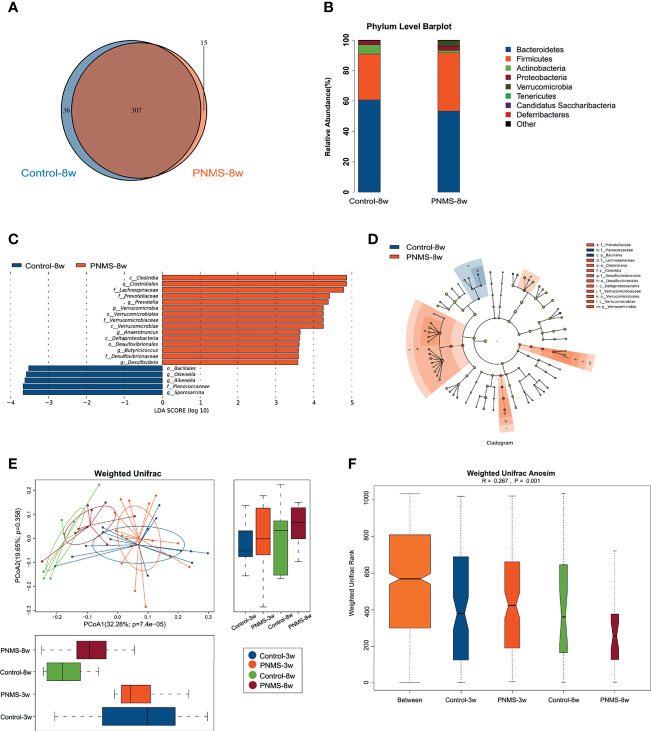
Prenatal maternal stress has a long-term impact on the gut microbiota in the offspring. **(A)** Venn diagram of the operational taxonomic units (OTUs). **(B)** Gut microbiota composition at the phylum level. **(C, D)** Linear discriminant analysis (LDA) scores and the cladogram showed significant bacterial differences between the two groups by linear discriminant analysis effect size (LEfSe). **(E, F)** The gut microbiota of the 3- and 8-week-old offspring were put together for weighted Unifrac principal coordinate analysis (PCoA) and weighted Unifrac analysis of similarities (ANOSIM). *PNMS*, prenatal maternal stress. Control-3w (*n* = 15) *vs.* PNMS-3w (*n* = 15) *vs.* Control-8w (*n* = 8) *vs.* PNMS-8w (*n* = 8).

To describe the long-term effects of PNMS on the gut microbiota, high-throughput sequencing was performed on the feces of 8-week-old offspring. The results showed that gut dysbiosis characterized by overgrowth of *Desulfovibrio* induced by PNMS continued in the 8-week-old offspring. The Venn diagram showed 370 OTU types in common, 52 OTU types that were exclusive to the control group, and 21 OTU types that were exclusive to the PNMS group (**Figure 6A**). The relative abundance of the bacterial phyla across the two groups showed no statistical difference (**Figure 6B**). Desulfovibrionales, Desulfovibrionaceae, and *Desulfovibrio*, which are associated with inflammation, persisted from 3 to 8 weeks and may play a key role in the susceptibility to colitis in adulthood (**Figures 6C, D**). PCoA at 3 and 8 weeks of age showed that the two groups of mice on a free diet after weaning tended to separate on the microbiota of the offspring (**Figure 6E**). Weighted Unifrac ANOSIM revealed that the differences among the four groups were significant (**Figure 6F**). In addition, the abundance of the pro-inflammatory genus *Prevotalla* increased in PNMS mice compared with that in the control mice at 8 weeks of age.

### PNMS Aggravated the Severity of Experimental Colitis in Adult Mice

**Figure 7:**
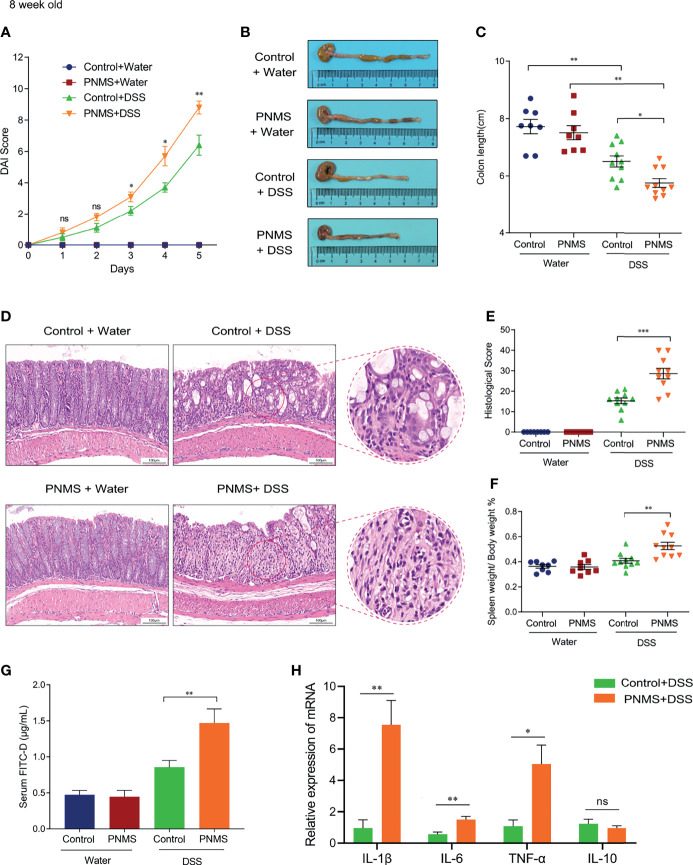
Prenatal maternal stress aggravated the severity of experimental colitis in adult mice. **(A)** Disease activity index (DAI) scores during the process. **(B)** Colon appearance after treatment. **(C)** Colon lengths. **(D)** Histopathological changes of the colon section detected by hematoxylin and eosin (HE) staining. **(E)** Histological scores of colonic inflammation. **(F)** Ratio of spleen weight to body weight. **(G)** Fluorescein isothiocyanate–dextran (FITC-D) intestinal permeability assay. **(H)** Relative mRNA expressions of the inflammation factors in the colon. *PNMS*, prenatal maternal stress; *DSS*, dextran sulfate sodium. *Scale bar*, 100 μm. Magnification, ×200. *p < 0.05, **p < 0.01, ***p < 0.001. Control+Water (*n* = 8), PNMS+Water (*n* = 8), Control+DSS (*n* = 10), PNMS+DSS (*n* = 10). ns, P < 0.05.

**Figure 8:**
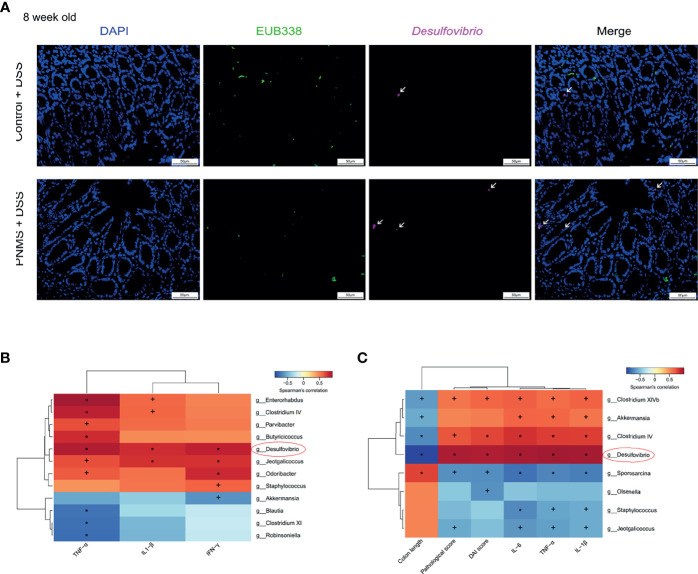
The relative abundance of *Desulfovibrio* increased in PNMS offspring mice and was associated with intestinal inflammation. **(A)** Fluorescence *in situ* hybridization (FISH) detection of *Desulfovibrio* in colon tissues in 8-week-old colitis model offspring. A fluorescein isothiocyanate (FITC)-conjugated universal bacterial 16S rDNA-directed oligonucleotide probe (EUB338, *green*) and a Cy5-conjugated *Desulfovibri*o specific probe (*red*) were applied. **(B, C)** Heatmap of Spearman’s correlations between the differential bacteria and markers of intestinal inflammation in 3- and 8-week-old offspring. *DSS*, dextran sulfate sodium; *PNMS*, prenatal maternal stress. *Scale bar*, 50 μm. Magnification, ×400. ^+^*p* < 0.05, *p < 0.01 (Spearman’s test).

The establishment of the gut microflora in early life and intestinal mucosal chronic inflammation have far-reaching impacts on intestinal health. In our study, we determined whether or not PNMS in early life increases the susceptibility to colitis in adult mice. Eight-week-old offspring were randomized to one cycle (5 days) of DSS treatment (2% in drinking water) or water treatment. The body weight and defecation of the offspring were recorded daily during the modeling process. Compared with the DAI scores in the normal control, the DAI scores in PNMS mice were significantly higher on the third, fourth, and the fifth day following modeling (**Figure 7A**). In mice without DSS treatment, no difference in the serum concentration of the marker FITC-D was found regardless of PNMS exposure (**Figure 7G**). Interestingly, in the colitis model groups, the mice exposed to PNMS showed higher FITC-D concentrations than did the control mice, suggesting higher intestinal permeability and more severe intestinal barrier destruction. The spleen/body weight ratio and the colon length of the PNMS and control mice were measured as indicators of immune/inflammation response. The colon lengths of the colitis model mice in both groups were shorter than those of the non-model mice (**Figures 7B, C**). Moreover, the colon lengths of the model mice exposed to PNMS were significantly shorter than those of the mice that were not, and the difference was statistically significant (**Figures 7B, C**). In addition, the spleen/body weight ratio showed changes similar to the colon length. In the colitis model groups, PNMS mice had a higher spleen weight-to-body weight ratio than did the control mice, suggesting higher levels of systemic inflammation (**Figure 7F**). Moreover, the histological sections of the colon in the PNMS group showed marked neutrophil and lymphocyte infiltration, crypt damage, and injury of the epithelial structures and had higher histopathological scores (**Figures 7D, E**). Subsequently, we detected the relative expression levels of the pro-inflammatory cytokines in the colitis model mice. Higher mRNA expression levels of IL-1β, IL-6, and TNF-α were observed in mice with PNMS (**Figure 7H**), whereas no statistical difference was found in the expression of IL-10. As expected, the highest proportion of positive *Desulfovibrio* probe was found in the colitis model mice exposed to PNMS (**Figure 8A**). Notably, a positive correlation was observed between the abundance of *Desulfovibrio* and the indicators associated with DSS-induced colitis (**Figure 8C**). In general, these findings suggested that PNMS can exacerbate the risk of colitis in adult offspring mice.

## Discussion

**Figure 9:**
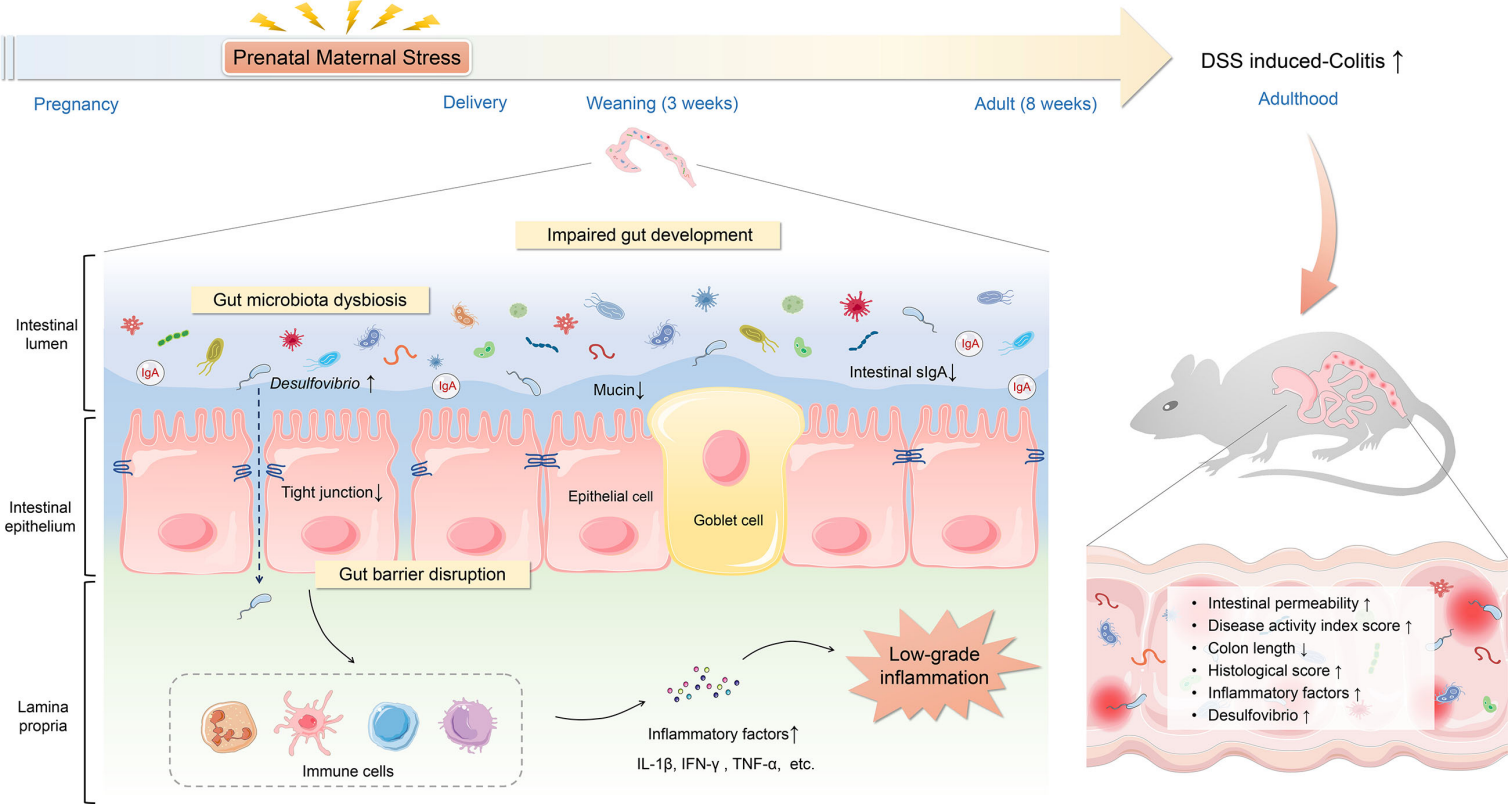
Schematic overview of how prenatal maternal stress increases the susceptibility to colitis in adult offspring. Prenatal maternal stress can impair the intestinal development, disturb the mucosal barrier function, induce low-grade inflammation, alter the composition of the microbiota in offspring, and increase the susceptibility to colitis in adulthood.

IBD incidence has been growing at an alarming rate. IBD is increasingly recognized as a serious public health concern worldwide. However, the pathogenesis of IBD is not thoroughly elucidated. Environmental factors, especially early life adversities, have profound and lifelong influences on gut health and contribute to the development of IBD. PNMS can induce far-reaching alterations in the composition of the gut microbiota and correlates with major physiological system outcomes (37, 44). To date, the role of PNMS in IBD susceptibility remains unclear. Our research focused on the influence of PNMS on intestinal growth and development, gut microbiota, gut barrier, and intestinal inflammation in offspring. We found that exposure to PNMS can impair normal intestinal development, disrupt the intestinal barrier function, induce microbiota dysbiosis characterized by persistent overgrowth of *Desulfovibrio*, and exacerbate experimental colitis of offspring in adulthood (**Figure 9**). These findings might refine the understanding of the potential relationship between early life stress and IBD.

Maternal stress is considered one of the important factors in the pathogeny of offspring stress dysregulation and is a prevalent symptom of neuropsychiatric diseases (45–47). Stress is closely related to IBD. The gut and brain possess a bidirectional communication *via* the microbiota–gut–brain axis, involving neural, endocrine, and inflammatory mechanisms. Stress exerts negative effects on the intestine structure, permeability, gut microbiota, and the mucosal immune system (12, 48). In the present study, we found a “heritable” stress-induced intestinal barrier damage phenotype characterized by an increased intestinal permeability and a decreased intestinal mucin production in the early stage of the next generation. The vertical transmission of the gut bacteria and the subsequent establishment of the microbiota in neonates overlap with the pivotal period of neurodevelopment and the establishment of intestinal immunity (49, 50). Therefore, an altered intestinal flora colonization caused by maternal stress may ultimately have negative downstream effects on the growth and development of the intestinal tract and the immunologic function in neonates. This imprinting may persist and mediate excessive immune activation and raise susceptibility to inflammatory diseases in adulthood. Jašarević et al. conducted RNA sequencing analyses of fetal intestines on day 18.5 of pregnancy between early prenatal stress (EPS)-exposed and control fetuses. EPS-induced alterations in the expressions of transcripts are related to normal gastrointestinal development, energy balance, and immune function in male fetuses (38). Our study found that PNMS has adverse effects on the gut development of mice in the early stage, but the difference was not observed in the adult stage. In addition, EPS caused progeny phenotypes with reduced weight gain, increased acute stress-induced corticosterone levels, and altered hypothalamic gene expression in male offspring. However, no obvious sexual differences with regard to weight changes and stress phenotype were observed in the pups exposed to mid-to-late maternal stress. The different results may be related to periods of the stress paradigm.

In terms of mucosal immunity, we observed that the sIgA production of PNMS pups significantly declined. These results supported evidence from clinical observations of a recent study that used a Canadian infant cohort and investigated the fecal sIgA concentrations of 1,043 infants and the maternal depression and stress symptom trajectories of their mothers. The data showed that infants born to mothers with antepartum depressive symptoms had lower levels of sIgA in their feces (51). Increases in corticosteroids induced by repeated restraint stress can reduce the sIgA secretion into the gut lumen (52). Glucocorticoids can be transferred to fetuses during pregnancy *via* the placenta, especially under stress (33), and this process may explain the lower intestinal IgA concentrations in the pups. The reduction of sIgA in an infant can negatively affect immune maturation and the crosstalk between microbes and sIgA (53), resulting in an upgrowth in the risks of *Clostridiodes difficile* colonization (54) and allergic conditions (55).

The early life stage represents a critical window of growth and development. The assembly of gut microbial communities during early life contributes to immune, endocrine, and other major developmental pathways (56). Many studies found that PNMS can alter the gut microflora in the next generations of human and animal models (36, 37, 44, 57). A cohort study of 56 Dutch healthy infants born vaginally showed that the infants of mothers with high cumulative stress (i.e., high reported stress and high cortisol concentrations) during pregnancy had significantly higher relative abundance rates of Proteobacteria groups known to contain pathogens (related to *Escherichia*, *Serratia*, and *Enterobacter*) and lower relative abundance rates of lactic acid bacteria (i.e., *Lactobacillus*, *Lactococcus*, and *Aerococcus*) and *Bifidobacterium*. Altogether, these characteristics indicate increased levels of inflammation (11, 36). Repeated restraint stress from embryonic day 14 to day 20 significantly reduced the abundance of *Lactobacillus* and increased the abundance of the *Oscillibacter*, *Anaerotruncus*, and *Peptococcus* genera in prenatal stress animals (37). Furthermore, maternal stress can alter the proteins related to vaginal immunity and the abundance of *Lactobacillus*, the prominent taxon in the maternal vagina. Loss of maternal vaginal *Lactobacillus* results in the decreased transmission of this bacterium to the offspring (44).

The period before weaning acts as a unique window of opportunity because pathological consequences appear later in life once the normal development of the immune system is perturbed (58, 59). Our study examined the intestinal microbiota of the offspring at 3 weeks of weaning and 8 weeks of adulthood. The results showed that PNMS induced a long-term gut microbiota dysbiosis characterized by the excessive growth of *Desulfovibrio*. Notably, Desulfovibrionales, Desulfovibrionaceae, and *Desulfovibrio* increased in 3- and 8-week-old PNMS mice. *Desulfovibrio*, one of the sulfate-reducing bacteria (SRB), may colonize the intestinal mucosa easily because of its ability to degrade the sulfated mucin of the mucus layer (60). SRB, which can metabolize genotoxic hydrogen sulfide (H_2_S), induces colitis in animal models and increases in abundance in patients with IBD (61, 62). A recent study has suggested that an increased intestinal permeability, inflammatory factors, and the intestinal H_2_S during weaning are responsible for the long-term pathological imprinting induced by excess calorie intake in early life and gut dysbiosis, leading to an increased risk of colitis in adulthood. The results of the verification experiment revealed that pathological imprinting can be prevented by the neutralization of IFN-γ and TNF-α, the production of hydrogen sulfide, or the normalization of intestinal permeability during weaning (17). In the present study, other pro-inflammatory bacteria, such as *Streptococcus* and *Enterococcus*, increased in 3-week-old PNMS offspring. However, Bifidobacteriales, Bifidobacteriaceae, and *Bifidobacterium* dramatically decreased in the 3-week-old pups with PNMS. Bifidobacteria are reliable indicators of a healthy microbiota in infants (63). Bifidobacteria in the infant gut can be directly acquired from the mother through vertical transmission (64). As a representative of the pioneer infant microbiome in the gut tract, *Bifidobacterium* species have a pivotal role in maintaining gut and immunity homeostasis, promoting intestinal development, and inhibiting the growth of pathogens (65, 66). In parallel, SCFA-producing bacteria, such as *Blautia* and *Robinsoniella*, showed significantly reduced abundance in 3-week-old offspring exposed to PNMS. SCFAs from the microbiome play a critical role in the crosstalk of the microbiota–gut–brain axis (67). The disruption of the brain–gut axis involves changes in neurotransmitters and is perhaps related to intestinal permeability, movement, and sensitivity. To clarify the role of the gut microbiota, we carried out FMT experiments. Interestingly, the feces of PNMS offspring induced low-grade intestinal inflammation and damaged the intestinal barrier function in the *in vivo* and *in vitro* experiments. Moreover, this phenotype can be partially reversed by the TNF-α inhibitor adalimumab. After treatment with adalimumab, no statistical difference in the indicators of inflammation and intestinal barrier function was observed between the two groups. This result suggested that blocking the inflammatory cytokines with neutralizing antibodies abolishes the effect of PNMS. We also found that the gut microbiota of the two groups of mice separated from their mothers during weaning did not tend to remain similar after receiving the same diet for 5 weeks. The intestinal microbiota dysbiosis induced by PNMS continued in the 8-week-old offspring, suggesting that the effect of PNMS on the progeny gut microbiota is persistent, profound, and tremendous. An increasing trend of *Desulfovibrio* and *Prevotella* was observed in 8-week-old adult mice. This trend was associated with chronic inflammatory disease. The pro-inflammatory intestinal microenvironment may persist and cause an indelible immunopathological imprint. Notably, the correlation analysis results showed that the relative abundance of *Desulfovibrio* was highly correlated with the level of inflammation in offspring mice, suggesting that the overgrowth of *Desulfovibrio* in early life is probably responsible for the development of inflammatory diseases later on. Overall, our data suggested that PNMS can induce low-grade intestinal inflammation and gut dysbiosis, is characterized by the increase of pro-inflammatory bacteria, especially *Desulfovibrio*, and the decrease of beneficial bacteria, and may produce long-term pathological imprint on the immune system and ultimately increase the susceptibility to colitis in adult offspring. The administration of antibiotics targeting *Desulfovibrio* or neutralizing antibodies in early life may provide a new perspective for the prevention of immune-inflammatory diseases.

Our study revealed that PNMS-exposed offspring were more prone to develop experimental colitis. The results have important implications for understanding how early life adversity influences intestinal health. However, potential limitations are present. This exploratory study did not involve experiments in which some perturbations were used to rescue the phenotypes. Future experiments can further define the precise mechanisms. Although no changes in intestinal permeability were detected in the two groups of mice in adulthood before the modeling, they showed disparities after colitis modeling. This result suggested that the gut microbiota is involved in this process. Our exploration of this hypothesis is in progress.

Overall, this study is the first to reveal that PNMS can impair gut development, disturb mucosal barrier function, and induce low-grade inflammation and overgrowth of *Desulfovibrio* in offspring, which contribute to the increased risk of IBD in adulthood. These results reveal a novel perspective on the long-term adverse impacts of maternal stress. Taken together, emotion-focused stress management strategies for mothers should be emphasized to prevent potential risk of IBD.

## Data Availability Statement

The raw data of 16S rDNA amplicon sequencing for this experiment has been uploaded to NCBI (SRA accession: PRJNA728691).

## Ethics Statement

The animal study was reviewed and approved by the Ethics Committee of Tianjin Medical University (Tianjin, China).

## Author Contributions

YS, RX, WL, KJ, and HC designed the research study. YS, RX, LL, GJ, HH, ML, YY, and BZ performed the experiments. YS, RX, LL, XL, and HC analyzed the data. YS, RX, and HC wrote the manuscript. BW, KJ, WL, XC, and HC made critical revisions. All authors who contributed to the design and writing of the paper agreed with the final version of the content of the manuscript.

## Funding

This research was supported by grants (82070545 and 81970477) from the National Natural Science Foundation of China and the Key Project of Science and Technology Pillar Program of Tianjin (20YFZCSY00020).

## Conflict of Interest

The authors declare that the research was conducted in the absence of any commercial or financial relationships that could be construed as a potential conflict of interest.

## Publisher’s Note

All claims expressed in this article are solely those of the authors and do not necessarily represent those of their affiliated organizations, or those of the publisher, the editors and the reviewers. Any product that may be evaluated in this article, or claim that may be made by its manufacturer, is not guaranteed or endorsed by the publisher.
