# Supplementary material for: Prenatal Maternal Stress Exacerbates Experimental Colitis of Offspring in Adulthood
Source: Front Immunol. 2021 Nov 3;12:700995. doi: 10.3389/fimmu.2021.700995 (PMC8595204; doi:10.3389/fimmu.2021.700995)
Supplement: Supplementary file 1 [file DataSheet_1.docx]

Supplementary Material

## Supplementary Figures
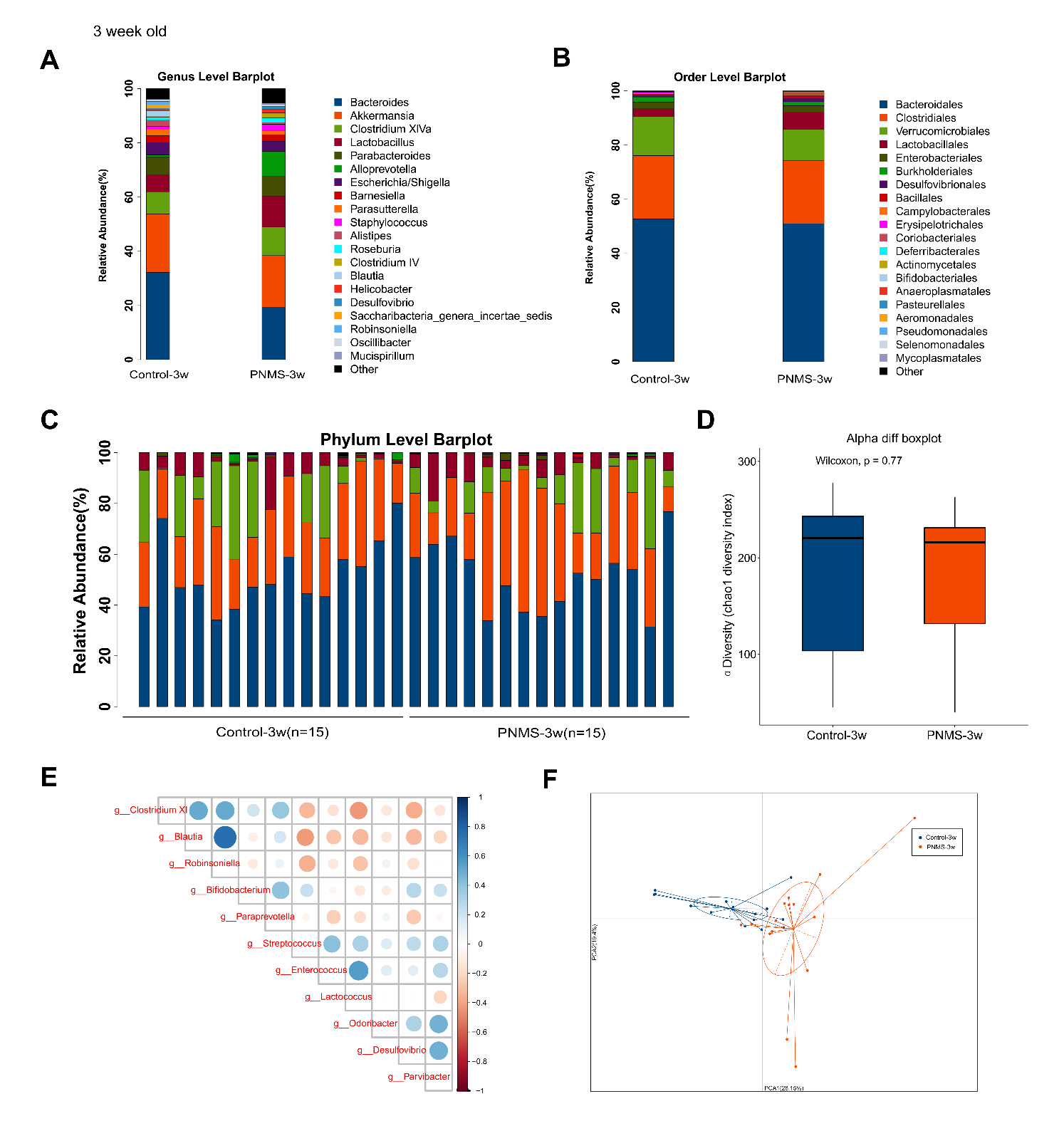


**Supplementary Figure 1.** Prenatal maternal stress altered the composition of gut microbiota in 3-week-old offspring. (A-B) The microbial composition at the level of bacterial genus and order. (C) Bar charts of the relative abundance of bacterial at the phylum level in each sample. (D) α-diversity was measured by chao1 diversity index. (E) Spearman correlation analysis of gut microbiota at the genus level. (F) Principal component analysis (PCA) of significantly different bacterial genera was performed. PNMS: Prenatal maternal stress. Control-3w(n=15) *vs* PNMS-3w (n=15).

**Supplemental Table**

**The Oligonucleotide primers used in Realtime-PCR analysis.**

| **Murine gene** | **Primer sequences** |
| --- | --- |
| mGAPDH | Forward primer: 5 -TGTGTCCGTCGTGGATCTGA-3 |
|  | Reverse primer: 5 -CCTGCTTCACCACCTTCTTGA-3 |
| mZO-1 | Forward primer: 5 -GGGCCATCTCAACTCCTGTA-3 |
|  | Reverse primer: 5 -AGAAGGGCTGACGGGTAAAT-3 |
| mClaudin3 | Forward primer: 5 -CCTGTGGATGAACTGCGTG-3 |
|  | Reverse primer: 5 -GTAGTCCTTGCGGTCGTAG-3 |
| mOccludin | Forward primer:5 -ACTATGCGGAAAGAGTTGACAG-3 |
|  | Reverse primer: 5 -GTCATCCACACTCAAGGTCAG-3 |
| mMuc2 | Forward primer:5 -TCGCCCAAGTCGACACTCA-3 |
|  | Reverse primer: 5 -GCAAATAGCCATAGTACAGTTACACAGC-3 |
| mCryptdin | Forward primer:5 -CAGCCGGAGAAGAGGACCAG-3 |
|  | Reverse primer: 5 -TAGCATACCAGATCTCTCAACGATTC-3 |
| mReg3γ | Forward primer:5 -TTCCTGTCCTCCATGATCAAAA-3 |
|  | Reverse primer: 5 -CATCCACCTCTGTTGGGTTCA-3 |
| mIL-1β | Forward primer: 5 -GTGGCTGTGGAGAAGCTGTG-3 |
|  | Reverse primer: 5-GAAGGTCCACGGGAAAGACAC-3 |
| mIFN-γ | Forward primer: 5-GCATCTTGGCTTTGCAGCT-3 |
|  | Reverse primer: 5-CCTTTTTCGCCTTGCTGTTG-3 |
| mTNF-α | Forward primer: 5-ACTCCAGGCGGTGCCTATG-3 |
|  | Reverse primer: 5-GAGCGTGGTGGCCCCT-3 |
| mTGF-β | Forward primer: 5-GCTGAACCAAGGAGACGGAAT-3 |
|  | Reverse primer: 5-GCTGATCCCGTTGATTTCCA-3 |
| mIL-6 | Forward primer: 5-CCAGTTGCCTTCTTGGGACT-3 |
|  | Reverse primer: 5-GGTCTGTTGGGAGTGGTATCC-3 |
| mIL-10 | Forward primer: 5-TGGACAACATACTGCTAACCG-3 |
|  | Reverse primer: 5-GGATCATTTCCGATAAGGCT-3 |
| hTNF-α | Forward primer: 5- AGCCCTGGTATGAGCCCAT CTATC-3 |
|  | Reverse primer: 5-TCCCAAAGTAGACCTGCCC AGAC-3 |
| hIL-1β | Forward primer: 5- ATGCACCTGTACGATCACTG-3 |
|  | Reverse primer: 5- ACAAAGGACATGGAGAACACC-3 |
| hClaudin3 | Forward primer: 5-CATCACGTCGCAGAACATCT-3 |
|  | Reverse primer: 5- GAGTCGTACACCTTGCACTG-3 |
